# Graphene-Based Spatial Light Modulator Using Metal Hot Spots

**DOI:** 10.3390/ma12193082

**Published:** 2019-09-21

**Authors:** Zhanshan Sun, Yuejun Zheng, Yunqi Fu

**Affiliations:** College of Electronic Science and Technology, National University of Defense Technology, Changsha 410072, China; szs199252@163.com (Z.S.); erikzhengyang@126.com (Y.Z.)

**Keywords:** Graphene, near infrared, spatial light modulator, hot spot

## Abstract

Here, we report a graphene-based electric field enhancement structure achieved by several adjacent metal nanoribbons which form the hot spots of the electric field and thus promote the absorption of the single layered graphene below the hot spots. Based on the tunability of the graphene’s Fermi level, the absorption rate can be modulated from near 100% to 35% under low electrostatic gating, leading to a 20 dB modulation depth of reflectance. Compared with the existing near infrared spatial light modulators such as optical cavities integrated with graphene and other structures utilizing patterned or highly doped graphene, our design has the advantages of strong optical field enhancement, low power dissipation and high modulation depth. The proposed electro-optic modulator has a promising potential for developing optical communication and exploiting big data interaction systems.

## 1. Introduction

Optical communication is highly demanded and widely used in networks and the Internet of Things (IOT) due to its advantages such as high channel capacity, anti-interference capability, good confidentiality, and low cost [1]. Specially, big data interaction in small satellites and unmanned aerial vehicles (UAVs) is rapidly needed because of the popularization of small near-earth satellites and UAVs. Since these applications tend to be smaller and lighter, communication systems are urgently required to have lower volumes and weights, as well as the ability to process big data within a short time. In contrast, microwave and millimeter wave communication systems are generally much bigger and heavier than optical systems, but they support a relatively narrow modulation bandwidth [2]. Additionally, current optical modulators using traditional optical components, like lens and spectroscopes [3], do not meet the requirements of miniaturization. Therefore, optical communication using nanoscale components and 2D materials has been proposed and is of great potential for prospective applications [4,5,6].

The spatial light modulator is one of the key components of optical communication [4,7]. Silicon-based spatial modulators show sufficient merits regarding device footprint, power consumption and fabrication. However, the weak electro-optic effect of silicon still sets a technical bottleneck for these devices [8]. Additionally, the existing platforms for light modulators that utilize liquid crystals [9], advanced materials exhibiting metal-insulator phase transitions [10], mechanically stretchable materials [11], or semiconductor interfaces [12], suffer from slow response speed, shallow modulation depth, and narrow bandwidth. In recent years, two-dimensional layered materials such as graphene, transition metal dichalcogenides (TMDs), black phosphorus and perovskite have emerged as alternative active materials for optoelectronic applications due to their exceptional optical and electronic properties [13]. Graphene, the best-known 2D material, has been widely used for many photonic and optoelectronic devices, operating at an extremely broad spectral range extending from the ultraviolet to microwave regions due to its unique linear energy–momentum dispersion relation. Its optical conductivity can be tuned by electrostatic gating or chemical doping [14]. This property has already been utilized to tune the absorption of light.

For absorptive modulators, several mechanisms have been proposed to achieve the effective absorption of incident light and realize the dynamic tunability of absorption rate or absorption bands by using graphene. Patterned graphene structures (e.g., ribbons, disks and stacked multilayer designs) [15,16], hybrid structures (e.g., graphene-gold nanoparticles and plasmonic waveguides) [17,18,19] and metamaterials (e.g., Fano resonance structures and integrated optical cavities) [8,20,21] have been used to enhance the light-matter response and tune the reflectance/absorbance of these devices [22,23,24]. However, because graphene is of low photoconductivity in the near-infrared region, it is hard to excite graphene’s intrinsic plasmonic resonance, so most of the mentioned works have introduced devices that operate at mid-infrared or terahertz wavelengths. Waveguides and microcavities have been proposed to realize the absorption of near infrared light, but most of these light-trapping strategies require electrolyte doping to change the graphene Fermi energy, and the modulation speed is thus limited to several kilohertz [8]. Meanwhile, these devices are usually based on highly nanostructured configurations, including highly patterned graphene or optical cavities composed of lots of dielectric layers [25]. At the near infrared wavelength, since the tunability of absorption property is not available for low-doped graphene, the high modulation depth (>10 dB) of most graphene-based near-infrared modulators depends on high electrostatic gating voltage or highly chemical doping [26]; as such, the power consumption and the difficulty of preparation inevitably increase.

Recently, graphene-metasurface plasmonic structures have been predicted to have the potential to achieve high modulation depth and speed [7]. This significantly enhanced light-matter interaction makes it possible to greatly promote the absorption rate of optical devices at the near infrared spectrum. Moreover, the relaxation time of plasmons is typically on a femtosecond level, which offers an opportunity for achieving an extremely fast modulation speed with graphene. Here, we designed an electric field enhancement structure using several tightly placed metal nanoribbons which build plasmonic resonance slits and form the light-trapping hot spots of an electric field. A high-mobility dual-layer graphene capacitor was used as a lossy medium and placed below the hot spots. The two graphene sheets operated with strong absorption to the incident light, and the absorption rate could be modulated using the Pauli blocking effect by shifting the graphene’s Fermi level over the threshold of interband transition through electrostatic gating. To reduce the gate voltage and maintain the high mobility of graphene, high-κ substrate tantalum pentoxide (Ta_2_O_5_) was used as the gate insulator; thus, the power dissipation could be significantly reduced, and a high modulation speed could be kept.

## 2. Materials and Methods

**Figure 1:**
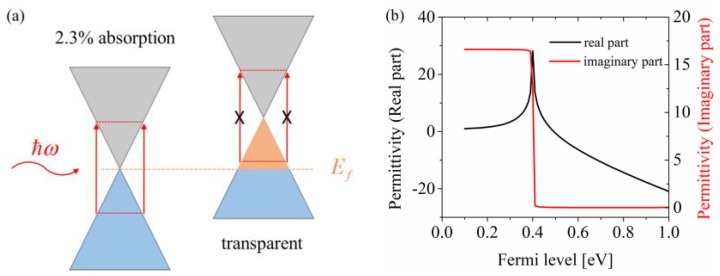
(**a**) Illustration of the Pauli blocking effect. (**b**) Real part and imaginary part of graphene’s permittivity with different Fermi levels.

Based on the Kubo formula [15,16,18], the conductivity of graphene is induced by its interband and intraband transitions:

(1) $$\sigma_{g}=\sigma_{intra}+\sigma_{inter}$$

(2) $$\sigma_{intra}=j\frac{q^{2}k_{B}T}{\pi \hbar (\hbar \omega +j\Gamma_{c})}[\frac{\mu_{c}}{k_{B}T}+2\ln(e^{-\mu_{c}/k_{B}T}+1)]$$

(3) $$\sigma_{inter}=j\frac{q^{2}}{4\pi \hbar }\ln[\frac{2|\mu_{c}|-(\hbar \omega +j\Gamma_{c})}{2|\mu_{c}|+(\hbar \omega +j\Gamma_{c})}]$$

where q is the charge of electron, ℏ is the reduced Planck constant, $k_{B}$ is the Boltzmann constant, *T* is the effective carrier temperature, *ω* is the optical frequency, and $\mu_{c}$ is the Fermi level of graphene. $\Gamma_{c}=q\hbar v_{F}{}^{2}/\mu \mu_{c}$ represents the damping constant, where $v_{F}\approx 10^{6}\;m\cdot s^{-1}$ is the Fermi velocity and $\mu \approx 10^{4}\;cm^{2}\cdot V^{-1}s^{-1}$ is the electron mobility. At the charge neutral point, the absorption rate of single layered graphene, which is determined by the fine structure constant $\alpha =q^{2}/\hbar c$, is 2.3% over a wide spectrum from the terahertz to visible wavelengths [25]. However, due to the Pauli blocking effect, as shown in Figure 1a,b, when the Fermi level $\mu_{c}>\hbar \omega /2$—the imaginary part of graphene’s permittivity—decreases rapidly, the interband transition of carriers is forbidden, and the absorption of photon gets quite weak, enabling a remarkable modulation. For the 1550 nm wavelength, the photon energy ℏω ≈ 0.8 eV, and thus the critical switching-off Fermi level, could be set as 0.4 eV.

**Figure 2:**
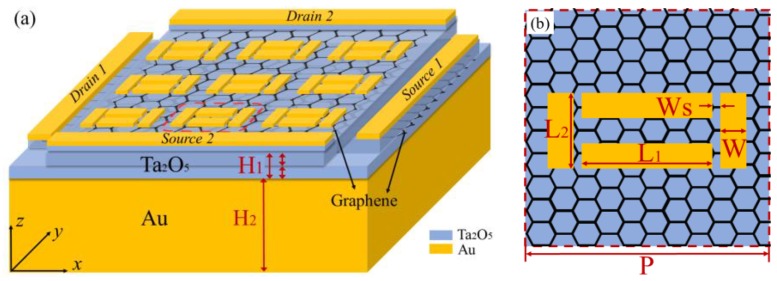
Structural parameters of the proposed modulator. (**a**) Configuration of the three dimensional structure with H_1_ = 40 nm, H_2_ = 100 nm. (**b**) Unit cell of the hot spots. The parameters are: P = 500 nm, L_1_ = 195 nm, L_2_ = 150 nm, W = 50 nm, Ws = 20 nm.

As shown in Figure 2, the hot spots were made up of four metal nanoribbons arranged in a dolmen shape. The slits between the transverse and longitudinal ribbons were designed to induce gap-plasmon resonance, which can trap the local electric field and boost the interaction of incident radiation with the graphene sheet. The graphene sheets were placed on a Ta_2_O_5_ substrate to form a grapheme-Ta_2_O_5_-graphene-Ta_2_O_5_ multilayered structure. The metal plate was deposited on the back of the substrate as the reflection mirror; thus, the absorption of the whole structure can be expressed as A = 1 − |S_11_|^2^, where |S_11_| is the reflection coefficient. The structure was investigated using 3D finite element method (FEM) software Comsol Multiphysics 5.4. Periodic boundary conditions were applied for the lateral boundaries, and perfectly matching layers (PML) were employed along the incident direction to eliminate the boundary scattering. The graphene could be defined by its bulk properties with the anisotropic permittivity:

(4) $$\varepsilon_{g}=\left[\begin{array}{ccc} \varepsilon_{xx} & & \\ & \varepsilon_{yy} & \\ & & \varepsilon_{zz} \end{array}\right],\;\varepsilon_{xx}=\varepsilon_{yy}=1+j\frac{\sigma_{g}}{\varepsilon_{0}\omega \Delta },\;\varepsilon_{zz}=2.5$$

where Δ = 0.34 nm is the thickness of graphene. However, because the graphene just has atomic thickness, the mesh generation is quite huge, so the simulation has to operate with large computation costs. Here, we set the graphene as a surface current density layer that can be defined as:

(5) $$J_{s}=\left[\begin{array}{ccc} \sigma_{g}\cdot E_{x} & & \\ & \sigma_{g}\cdot E_{y} & \\ & & 0 \end{array}\right]$$

To verify the absorption performance, graphene sheets under room temperature and low-doping ($\mu_{c}$ < 0.4 eV for 1550 nm) were firstly considered. Here, we assumed that $\mu_{c}=0.1\;eV$ and T=300 K. The metal nanoribbons and reflection mirror were both gold and defined by the Drude model: $\varepsilon (\omega )=\varepsilon_{\infty }-\omega_{p}{}^{2}/(\omega^{2}+i\gamma \omega )$ with $\varepsilon_{\infty }=\;1$, plasma frequency $\omega_{p}=\;1.37\;\times \;10^{16}\;s^{-1}$, and damping constant $\gamma =\;4.05\;\times \;10^{13}\;s^{-1}$. To adjust the absorption peak to 1550 nm, the geometric parameters of the proposed structure were set as: The period of unit cell P = 500 nm, the length of the nanoribbon in x-direction L_1_ = 195 nm, the length of the nanoribbon in y-direction L_2_ = 150 nm, the width of nanoribbon W = 50 nm, the width of gap Ws = 20 nm, the height of Ta_2_O_5_ substrate H_1_ = 40 nm, and the thickness of the metal mirror H_2_ = 100 nm, and the thickness of the nanoribbons *h* was 30 nm. It should be noted that the thickness and length of metal parts have a key role on the plasmonic behavior of the structure [27]. The refractive index of Ta_2_O_5_ substrate was 2.1 RIU (refractive index unit). Due to the high-κ of Ta_2_O_5_, the tuning gate potential was greatly reduced compared to the commonly used SiO_2_/Si insulator, which was determined by:

(6) $$V_{g}=\frac{2qd_{s}}{\pi \hbar v_{F}{}^{2}\varepsilon_{0}\varepsilon_{s}}[{\left(k_{B}T\right)}^{2}\int_{-\mu_{c}/k_{B}T}^{\mu_{c}/k_{B}T}\frac{x}{e^{x}+1}dx+k_{B}T\mu_{c}\ln(e^{\frac{\mu_{c}}{k_{B}T}}+1)+k_{B}T\mu_{c}\ln(e^{-\frac{\mu_{c}}{k_{B}T}}+1)]$$

where $d_{s}$ is the thickness of insulator substrate, $\varepsilon_{0}$ is the free space permittivity, and $\varepsilon_{s}$ is the permittivity of Ta_2_O_5_. In this encapsulated graphene structure, the two graphene layers were gated reversely so the top graphene is n-doped while the bottom one is p-doped. This means that these two sheets have opposite Fermi levels (+$\mu_{c}$ and −$\mu_{c}$, respectively). Therefore, each one of the graphene sheets can serve as a gate and supply gate voltage to another. As a result, the applied gate voltages were about 3 V for 0.5 eV and 12 V for 1 eV. Thus, the dual layered graphene configuration could reduce power consumption and avoid the breakdown of insulator material.

**Figure 3:**
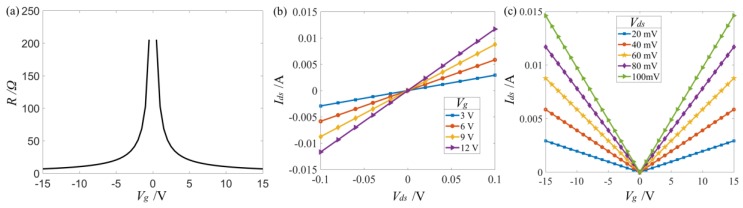
Theoretical electrical characteristics of graphene. (**a**) Resistance as a function of gate voltage. (**b**) Drain-source current as a function of drain-source voltage under different gate voltages (*V_g_*). (**c**) Drain-source current as a function of gate voltage under different drain-source voltage (*V_ds_*).

The theoretical electrical characteristics of graphene were studied and are shown in Figure 3, where *V_g_* is gate voltage, *R* is resistance of graphene, and *V_ds_* and *I_ds_* are drain-source voltage and current, respectively. At the charge neutral point, the theoretic minimum conductivity of graphene $\sigma_{\min}$ = 0. However, in practice, it was not zero when *V_g_* = 0 V due to charged impurities or charged puddles [28]. Besides, $\sigma_{\min}$ may not correspond to the minimum *V_g_*. Thus, the electronic properties of fabricated graphene will be different. To the best of our knowledge, $\sigma_{\min}$ is usually set to be $4q^{2}/h\approx 1.55\times 10^{-4}\;\Omega^{-1}$ in the simulation, so the maximum resistance $R_{\max}=1/\sigma_{\min}\approx 6.45\;k\Omega$ by ignoring the contact resistance of electrodes [29].

## 3. Results

**Figure 4:**
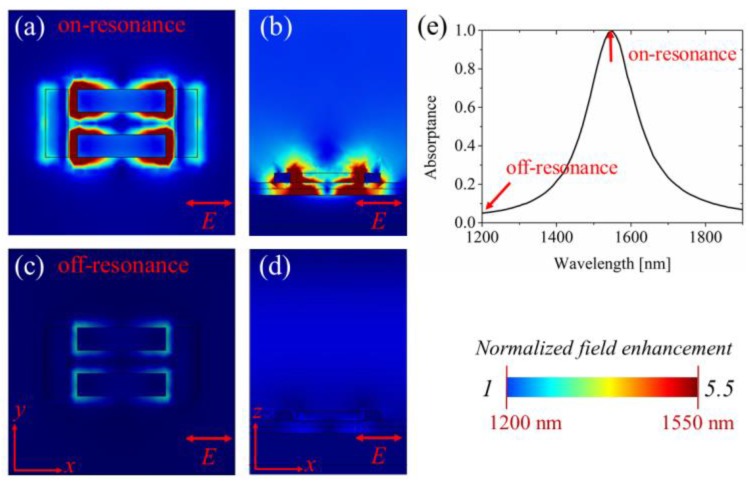
Intensity of the electric field at on-resonant wavelength and off-resonant wavelength. (**a**) The electric field of the incident light was trapped among the slits at resonant wavelength due to the coupling between the tightly placed nanoribbons. (**b**) For off-resonant wavelengths, the coupling effect could not be excited, so the electric field enhancement was not available. (**c**) Side view of the whole structure. At the on-resonant wavelength, the optical filed was concentrated surrounding the slits and absorbed by graphene, causing very few reflected waves. (**d**) At the off-resonant wavelength, more reflected waves were excited. (**e**) Illustration of on- and off-resonances.

In order to reveal physical insight, we investigated the absorbance of the proposed structure under the normal illumination of near-infrared light, whose electric field is parallel to the long nanoribbon (x-axis). Due to the presence of the metal back mirror, the minimum reflection from the hot-spot structure corresponded to the maximum absorption at the resonance wavelength. Based on the metasurface theory [30], the absorption of the incident wave can be attributed to the matching of the wave impedance between air and the artificial structure. Surface electric currents excited by the incident wave induce the superposition of inverse optical fields, which further contribute to the suppression of the reflection due to the electromagnetic coupling of optical fields at the air-nanoribbons interface. [17]. Therefore, the absorption enhancement of the graphene layer in the proposed structure can be illustrated by displaying its electric field distribution. For the resonant wavelength, the incident light excited the localized plasmonic resonance at the corners of the dolmen and induced strong coupling between adjacent nanoribbons, as shown in Figure 4a. As a result, it can be observed from Figure 4b that the electric field surrounding the slits of gold nanoribbons was concentrated and enhanced. The incident electric fields were trapped among the substrate layer as the guided gap-plasmon mode and induced the effects of near field enhancement and light energy concentration. As a consequence, the electric field among the slits was enhanced enough to stimulate the sufficient intraband and interband transition of graphene’s carriers. In contrast, for the off-resonant wavelength (1200 nm here), as shown in Figure 4c,d, there was little enhanced optical near field for absorption enhancement in graphene because this wavelength was far away from the gap-plasmon resonance wavelength on the spectrum. Therefore, the electric field could not be trapped, and the carrier transition was not available, so most of the incident light was reflected back to space.

**Figure 5:**
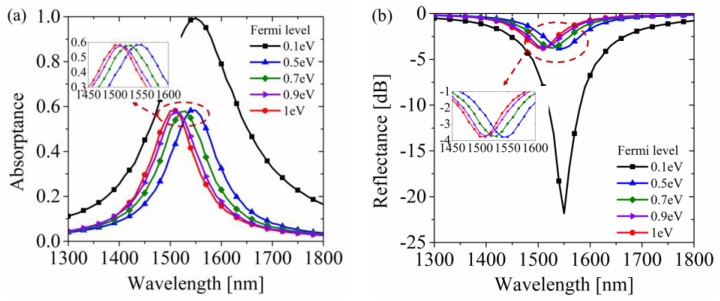
Absorbance and reflectance performances under different Fermi levels. (**a**) Absorbance of the proposed modulator. For low doping graphene (0.1 eV here), the absorbance peak occurred at a 1550 nm wavelength and reached near 100%. In contrast, the absorbance peak fell to 58% as the graphene was gated to 0.5 eV due to the Pauli blocking effect. The reflectance kept falling with the increase of the Fermi level. (**b**) Reflectance lines corresponding to the absorbance properties. The maximum modulation depth reached 20 dB at the 1550 nm wavelength.

To verify the modulation performance, the two graphene layers were gated to ±0.1 eV and ±0.5 eV. It can be seen from Figure 5a that the absorbance of the proposed structure was significantly promoted up to near 100% at 1550 nm under the ±0.1 eV Fermi level. In contrast, the absorption rate at 1550 nm reduced to 58% under the ±0.5 eV Fermi level due to the blocking effect of interband transition. As a result, it can be observed from Figure 5b that the modulation depth of reflectance was up to 18 dB from −21.9 dB to −3.8 dB, which was large enough for the practical application of spatial light modulator [4]. With the increase of the Fermi level, the absorption peak took blue-shift. The shifting mechanism on macroscopic scale was that the graphene with different Fermi levels had different electromagnetic properties regarding permittivity. The change of permittivity influenced the impedance matching as well as the resonant condition of the whole structure. The real part and imaginary part of graphene’s permittivity with different Fermi levels under a 1550 nm wavelength illumination are plotted in Figure 1b. This shows that after the critical switching-off Fermi level (about 0.4 eV), the real part decreased as the Fermi level increased, while the imaginary part remained essentially unchanged. Because the real part determined the phase response and the imaginary part determined the loss part, the intensity of the absorbance peak remained about the same, but the location took blue-shift. Therefore, although the Fermi level exceeded the threshold of the Pauli blocking effect, the modulation depth continued to increase, so the modulation depth reached 20 dB when the Fermi level was gated to ±1 eV.

**Figure 6:**
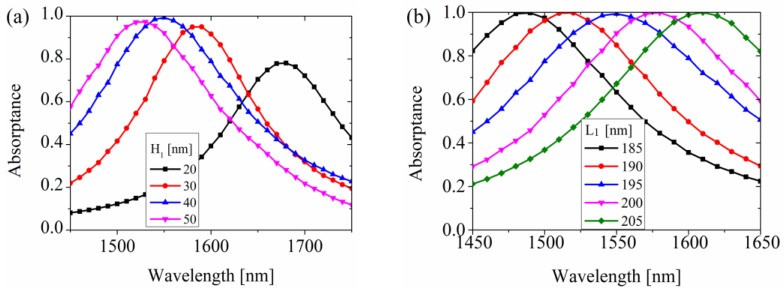

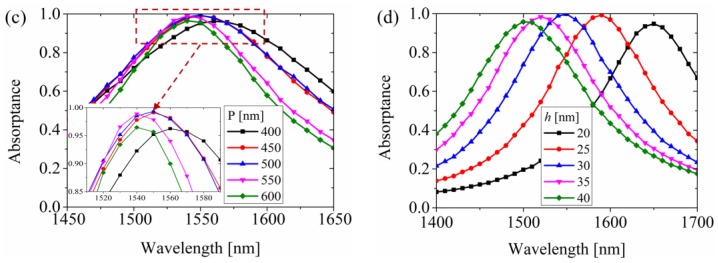
Influence of geometric parameters of the structure to the working wavelength. (**a**) The thickness of Ta_2_O_5_. (**b**) The length of the nanoribbon. (**c**) The period of the unit cell. (**d**) The thickness of the nanoribbon.

Next, we investigated the influence of geometric parameters of the structure on the working wavelength. Here H_1_, L_1_, P and *h* were considered. As shown in Figure 6, the electric field was most sensitive to the length and thickness of the nanoribbon, while the absorption rate remained nearly unchanged. Therefore, one can easily change the working spectrum by adjusting these parameters.

**Figure 7:**
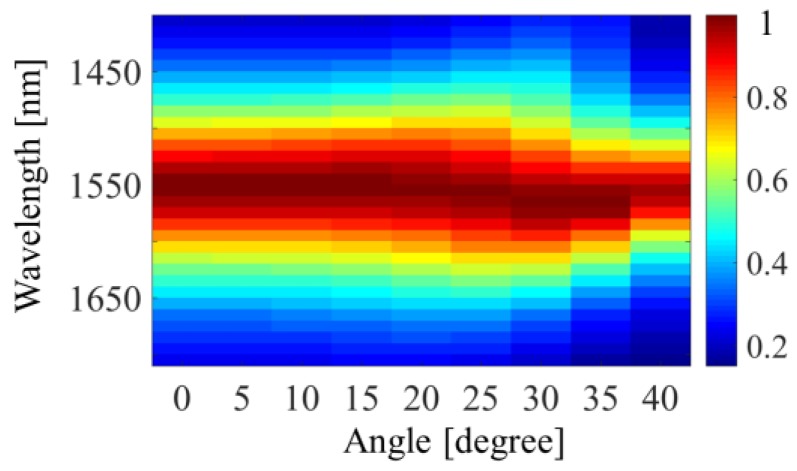
Absorbance diagram of the proposed modulator as a function of the free space wavelength and angle of incidence.

The discussion above is based on the normal incident light, but in practice, manipulating incident light with oblique angles is one of the important indexes for spatial light modulators. We next demonstrated the absorbance diagram of the proposed modulator as a function of the free space wavelength and angle of incidence, as shown in Figure 7. For TM-polarized light, whose magnetic field was always parallel to the structure’s surface, it can be seen that the absorbance at 1550 nm remained higher than 90% when the incident angle reached 40°. This is because the gap-plasmon was not sensitive to the angle of incident light. As the incident angle kept increasing, the maximum absorption rate got smaller, and the absorption bandwidth became narrower. 

## 4. Conclusions

In summary, we have investigated the absorbance and modulation properties of hot-spot structures which were formed by tightly adjacent metal nanoribbons. Strong coupling occurred when the electric field of the incident light was trapped among the slits at the resonant wavelength. As the local electric field enhancement was excited, the energy of the incident light could be absorbed by the low doping graphene layer. The modulation performance was achieved by utilizing the Pauli blocking effect, which disabled the interband transition of the carriers with the high Fermi level. Our research reveals the field enhancement effect of hot spot structures and provides a guide for designing spatial light modulators at the near infrared spectrum applied in the interaction of UAVs and low earth orbiting satellites (LEOS).
